# Comment on: Serum cholesterol and LDL-C in association with level of diastolic blood pressure in type 2 diabetic patients

**DOI:** 10.12861/jrip.2012.06

**Published:** 2012-01-01

**Authors:** Hamid Nasri

**Affiliations:** ^1^Department of Nephrology, Division of Nephropathology, Isfahan University of Medical Sciences, Isfahan, Iran

**Keywords:** Cholesterol, Blood pressure, Dyslipidemia, Type 2 diabetes, Hypertension, Diabetic nephropathy, Lipids

**Implication for health policy/practice/research/medical:**

Monitoring of blood pressure and serum lipids would be beneficial for diabetic patients in preventing the disease progression especially diabetic nephropathy in this population.

There is increasing prevalence of hypertension worldwide (1). Hypertension is an important risk factor for cardiovascular disease and renal disease which are the leading cause of death in the world (1). Hypertension also is extremely common disease found in patients with diabetes mellitus. Indeed eighty to 90% of patients with type 2 diabetes mellitus (T2DM) will eventually develop hypertension, and about 20% of hypertensive patients develop diabetes (2). Hence, It is, therefore, very important to prevent progression of hypertensionin diabetes through appropriate treatment and monitoring (1). Dyslipidemia is also a major risk factor for macro-vascular complications in patients with T2DM (3). In the study of Behradmanesh *et al*. on 60,T2DM patients, a significant inverse correlation of serum cholesterol and LDL-C with level of diastolic blood pressure was observed. This study shows the influence of serum lipids on the development of hypertension and further support the strict control of dyslipidemia, as a one of the factors aggravating hypertension and resultant nephropathy (4).Maharjan *et al*. recently studied association of hypertension with microalbuminuria and lipid profile (2). They examined 130 hypertensive and 100 normotensive individuals of age> or = 25 years. They found a significant association of hypertension with microalbuminuria and dyslipidemia, microalbumin/creatinine ratio, low density lipoprotein, high density lipoprotein, triglyceride. They also found, waist circumference, body mass index and waist hip ratio, and were significantly higher in hypertensive than in normotensive persons. They concluded that higher lipid levels, in hypertensive patient are a established risk factor for progressing into diabetes and cardiovascular diseases (2). Previously in a group of T2DM, we observed the association of serum lipoprotein (a) with hypertension in diabetic patients (5). Also the positive relationship between serum HDL-cholesterol, LDL-cholesterol and systolic blood pressure in patients with T2DM was also reported in our another study (6). However it is also possible that, poor glycemic control, prolonged duration of diabetes and coexisting hypertension aggravates dyslipidemia in T2DM and make a vicious circle to increase the blood pressure too (3). Hence, monitoring of these parameters would be beneficial for diabetic patients in preventing the disease progression in this population.

## Author’s contribution

HN is the single author of the manuscript.

## Conflict of interests

The author declared no competing interests.

## Ethical considerations

Ethical issues (including plagiarism, data fabrication, double publication) have been completely observed by the author.

## Funding/Support

None.
